# A Case of Pneumomediastinum and Pneumoperitoneum with Concurrent Massive Subcutaneous Emphysema due to Repositioning of a Tracheostomy Tube

**DOI:** 10.7759/cureus.3881

**Published:** 2019-01-14

**Authors:** Karim O Elkholy, Hamza Akhtar, Eric Landa, Yury Malyshev, Sonu Sahni

**Affiliations:** 1 Internal Medicine, Brookdale University Hospital Medical Center, New York, USA; 2 Internal Medicine, Ross University School of Medicine, Bridgetown, BRB

**Keywords:** pneumomediastinum, tracheostomy, mechanical ventilation, tracheal tube, subcutaneous emphysema, pneumoperitoneum, spontaneous pneumoperitoneum, spontaneous pneumomediastinum, ventilator management

## Abstract

Tracheostomy is a common procedure seen in critically ill patients that require long term ventilatory support. As with all airway access procedures, tracheotomy with prolonged tracheal tube placement comes with possible risks such as tracheal scarring, tracheal rupture, pneumothorax, tracheoesophageal fistula among others. Another possible complication, though rare, is escape of free air into the surrounding tissue, as well as pneumomediastinum (PM). This may occur due to various reasons, some of them being tracheal rupture, barotrauma or tracheal tube mispositioning. Pneumomediastinum may present with concurrent free air in other body cavities such as the peritoneum, thorax or subcutaneous tissue. Though often not life-threatening it may require treatment including high flow oxygen, ventilator management or occasionally, surgical intervention. Herein we describe a rare case of PM with communicating pneumoperitoneum and massive subcutaneous emphysema due to tracheal tube mispositioning along with a review of the literature.

## Introduction

Tracheostomy is often performed on critically ill patients usually after a prolonged period of mechanical ventilation via an endotracheal tube to facilitate weaning, improve patient comfort, and allow safe discharge from intensive care units [1]. Complications from the presence of prolonged tracheostomy tubes include subglottic and tracheal stenosis, granulation tissue formation, tracheal wall depression and rupture (fracture), tracheomalacia, tracheoesophageal and tracheoinnominate fistulas [2]. Pneumomediastinum (PM), also referred to as mediastinal emphysema, is defined as the presence of free air in the mediastinal cavity. Its etiology may be a result of trauma or caused iatrogenically due to endoscopic or other therapeutic procedures [3]. It may also be spontaneous in the setting of physiologic processes or other chronic pulmonary conditions [4]. PM may present independently or depending on etiology may present concurrently with pneumoperitoneum and other manifestations of free air [5]. Herein we present a case of PM, pneumoperitoneum and massive subcutaneous emphysema in the setting of manipulation and mispositioning of a tracheostomy tube.

## Case presentation

**Figure 1:**
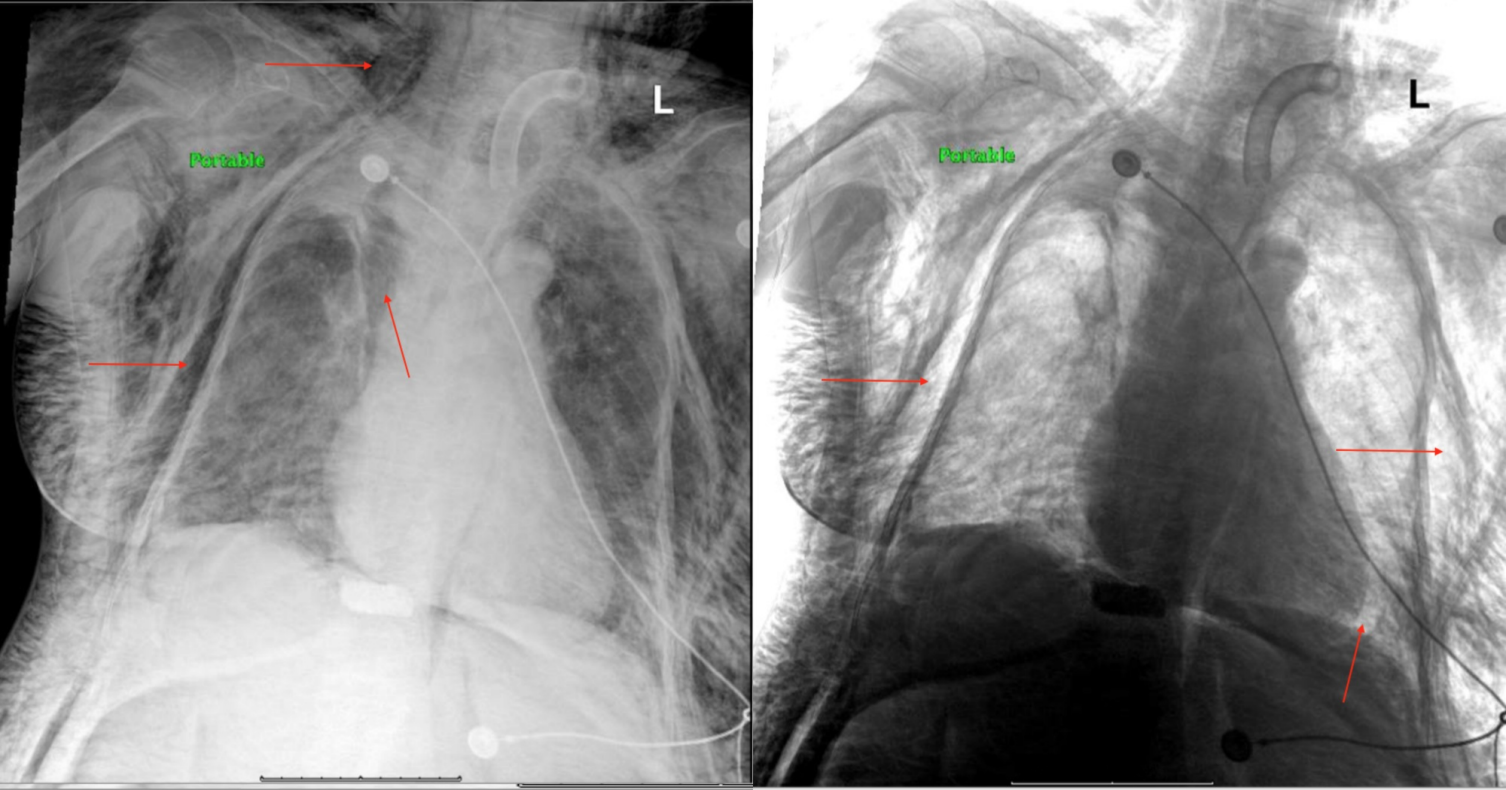
Portable chest X-ray showing pneumomediastinum and massive subcutaneous emphysema. Left: Chest X-ray showing free air in the subcutaneous tissue of neck and chest as well as mediastinum. Right: Inverted image of chest X-ray showing free air in the subcutaneous tissue of the chest and around the heart. Arrows point to the presence of free air in the subcutaneous and the mediastinum.

An 84-year-old female, nursing home resident, with a past medical history of type II diabetes mellitus, hypertension, cerebrovascular accident, anoxic brain injury with permanent percutaneous endoscopic gastrostomy (PEG) tube and tracheostomy to ventilator was noticed to have a high peak pressure on the ventilator on a routine check by the nursing home respiratory therapist. Suctioning was attempted but was unsuccessful. The tracheostomy tube was changed and during the process, increased resistance was felt. Soon thereafter it was noticed that blood was leaking from around the tracheostomy tube. Emergency medical services were called and the patient was brought to the emergency department. On arrival the patient was found to be hypertensive with a blood pressure of 160/74 mmHg, heart rate of 80 beats per minute, respiratory rate of 18 breaths per minute, O_2_ saturation was 100% on 100% FiO_2_ fraction of inspired oxygen (FiO_2_) on assist control-volume control + ventilator mode and a temperature of 37.8°C. On examination, the patient appeared to be generally edematous and was found to have crepitus on palpation starting at the forehead proceeding all the way down to the chest, abdomen, pelvis and upper thighs. In addition, her upper arms bilaterally also appeared swollen with crepitus present on palpation. Examination of the head and neck revealed a tracheostomy with tracheal tube in place with slightly pink secretions and what appeared to be dried blood on gauze surrounding the tube. On auscultation of the chest Hamman’s crunch, a crunching sound, synchronized with the heartbeat was present, as well as slightly decreased breath sounds at the right apex. Stat chest X-ray was performed which revealed extensive PM associated with subcutaneous emphysema in the neck and right chest wall (Figure 1).

**Figure 2:**
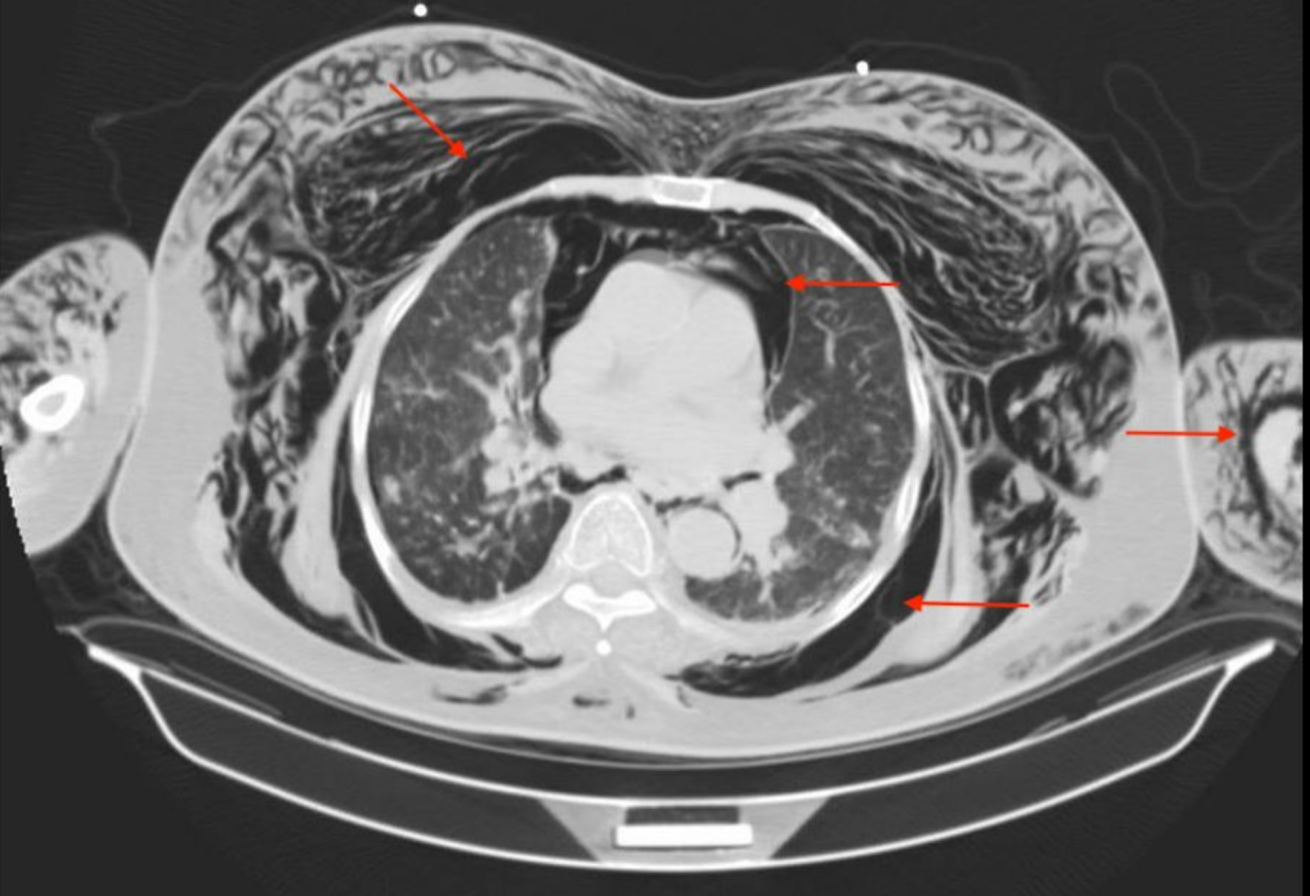
Computed tomography (CT) of the chest showing the presence of pneumomediastinum and subcutaneous emphysema. Arrows point to the areas of free air surrounding the heart and great vessels, massive subcutaneous emphysema of the thorax as well as upper extremities.

**Figure 3:**
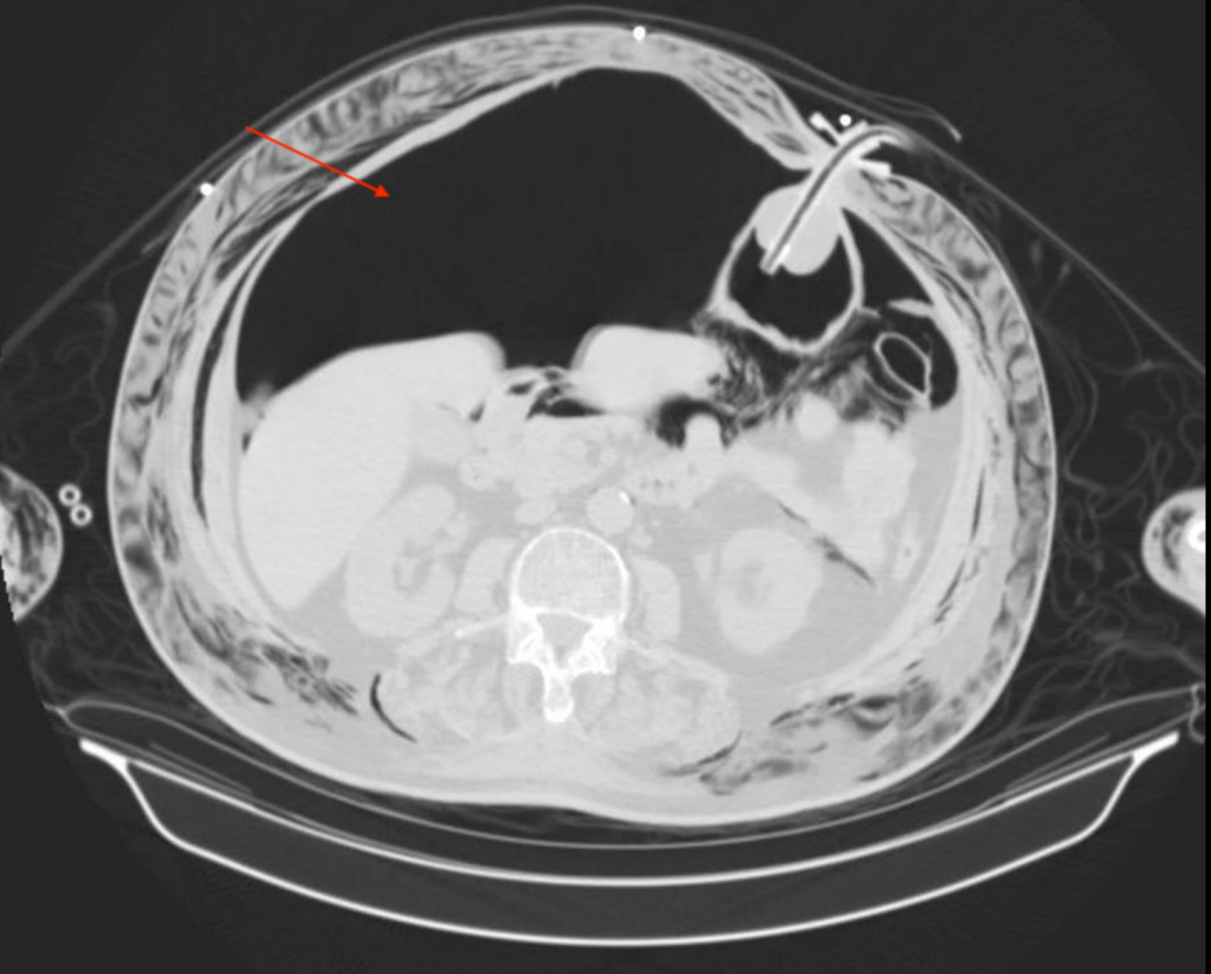
Computed tomography (CT) of the abdomen showing pneumoperitoneum. Arrow points to a significant amount of free air in the peritoneal cavity with posterior movement of the viscera, PEG tube in place. PEG: Percutaneous endoscopic gastrostomy

**Figure 4:**
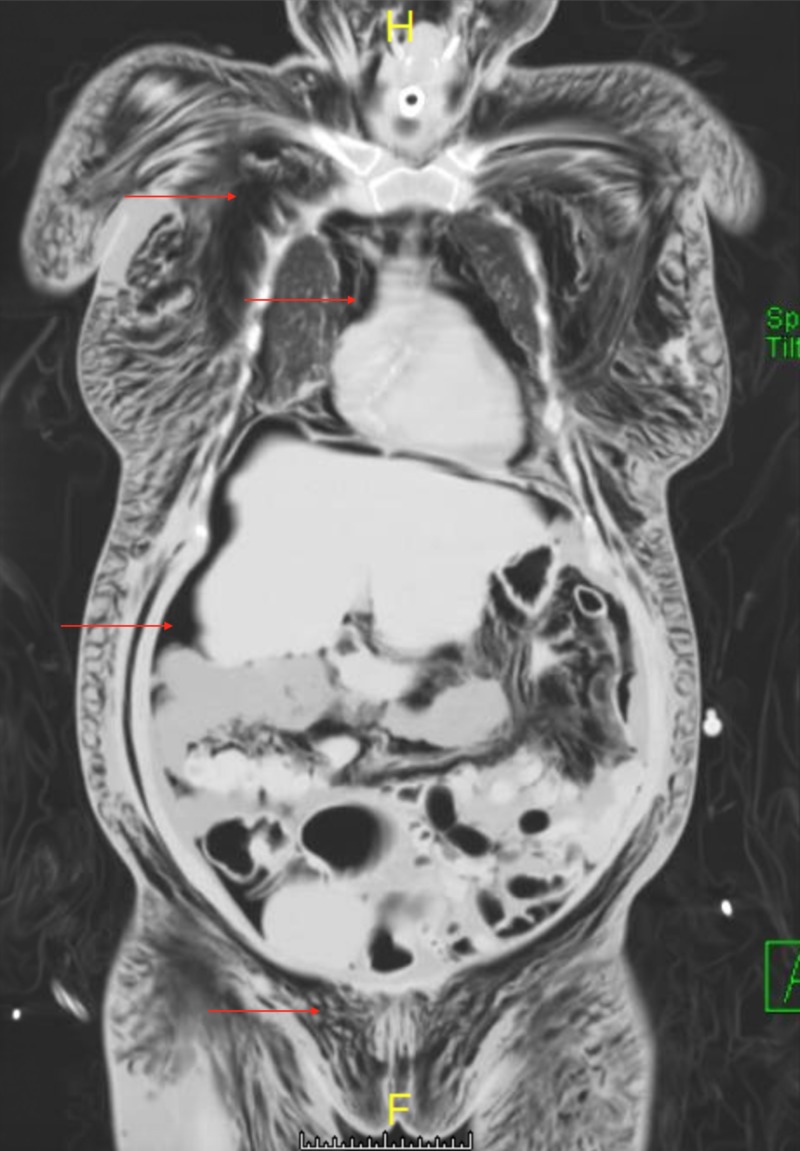
Coronal plane computed tomography (CT) of the chest, abdomen and of the pelvis showing the presence of pneumomediastinum, pneumoperitoneum and subcutaneous emphysema. Arrows from top to bottom show the presence of free air in the subcutaneous tissue of the chest wall, the mediastinum, sub-diaphragmatic air in the peritoneum and the subcutaneous tissue of the pelvis.

The patient was emergently assessed by an otolaryngologist and tracheoscopy was performed through the stoma with the tracheal tube in place. It was determined the tracheal tube was in proper position and no obvious tear or leakage in the tracheal wall was noted. Further imaging was warranted and a computed tomography (CT) of the chest, abdomen and pelvis was conducted. CT of the chest showed what appeared to be a 7-mm linear gas density projecting from the posterior wall of the upper trachea which was not seen on tracheoscopy. In addition, it also showed extensive subcutaneous emphysema of the neck and thoracic walls, extensive PM, gas density surrounding the lungs within both the pleural and extra-pleural spaces, measuring 10 mm in the anterior lower right hemithorax and less than 10 mm in the anterior lower left hemithorax (Figure 2). CT of the abdomen and pelvis showed large pneumoperitoneum with posterior displacement of the viscera, numerous gas densities in the mesenteric fat and retroperitoneum, extensive subcutaneous emphysema in the soft tissues extending to the level of the labia and anterior thighs bilaterally (Figures 3, 4).

For further monitoring, the patient was transferred to the medical intensive care unit (MICU). Lab results at the time of admission and discharge from the MICU are displayed (Table 1).

**Table 1:** Lab results from admission to MICU and upon discharge from MICU. BUN: Blood urea nitrogen; MICU: Medical intensive care unit

	Reference values	On admission	On discharge
White Blood Cells	(4.50-10.20) x 10^9^/L	16.2	15.7
Hemoglobin	11.4-15.5 g/dL	8.3	7.7
Hematocrit	37.0-43.7%	27.3%	28.8%
Platelets	(180-401) x 10^9^/L	424	393
BUN	7.0-17.0 mg/dL	35.0	38.0
Creatinine	0.52-1.04 mg/dL	0.53	0.72
Sodium	133-145 mEq/L	134	149
Potassium	3.5-5.1 mEq/L	4.0	4.7
Chloride	98-107 mEq/L	83	103
pH, arterial	7.35-7.45	7.38	7.41
HCO_3_, arterial	22.0-26.0 mmol/L	37.7	31.6
pCO_2_, arterial	35.0-45.0 mmHg	65.1	51.4
O_2_ sat, arterial	96.0-97.0%	98.5%	96.2%

The patient remained on the ventilator through the tracheostomy at 100% FiO_2_ with a positive end expiratory pressure (PEEP) of 0 cmH2O. Due to the substantial pneumoperitoneum, there was concern for abdominal compartment syndrome. Bladder pressure was assessed which revealed a pressure of 11 mmHg (normal range: 0-15 mmHg). Repeat chest X-ray 12 hours later showed a slight improvement in subcutaneous emphysema as less free air was evident in the soft tissue. The patient had episode of fever and was treated with cefepime for possible pneumonia. She remained hemodynamically stable and was transferred to telemetry floor for continuing cardiac monitoring. While on the floor the patient continued to receive antibiotics and remain on ventilator support. Five days later on nursing rounds, she was found to be unresponsive without a pulse. She was assessed and found to be deceased.

## Discussion

Herein we describe a case of 84-year-old woman diagnosed with PM, pneumoperitoneum and massive subcutaneous emphysema due to misadjusting of a tracheal tube. It appears likely that in our patient there may have been some point of mechanical insult leading to free air entering the subcutaneous tissue, mediastinum, and peritoneum. From imaging it was difficult to determine the exact point of possible insult or origin of air penetration. It is unclear what was causing the increased peak pressure that prompted the adjustment of the tracheal tube. In general, complications associated with tracheostomies are dependent on the time elapsed from the procedure with early complications being related to the immediate surgery including posterior tracheal wall injury, subcutaneous emphysema, tube dislodgement and tracheal tube obstruction [2]. Later complications are fairly common and can occur in up to 65% of patients which may also include tube mispositioning, dislodgement, tracheal stenosis or subcutaneous emphysema [6]. In addition, injury to the tracheal wall from high cuff pressure or improper positioning of the tube can lead to ulceration and formation of fistulas with nearby structures such as tracheoesophageal fistula and tracheoinnominate artery fistula [6]. Tracheostomy tube misposition is a late complication which can occur in up to 10% of patients [1]. The most common malposition is occlusion of the tracheostomy tube by the posterior tracheal wall which has been noted in up to 92% of cases [1]. This is in contrast to tracheostomy dislodgement which is a life-threatening event with a mortality rate that can be as high as 50% [7]. The tracheal tube termination should be approximately two to three centimeters above the carina as with endotracheal tubes. Anatomically at this depth the trachea is passing through the mediastinum. Disruption of the tracheal wall in this location may predispose air leaking into the mediastinal space leading to pneumomediastinum as we saw in our patient.

**Figure 5:**
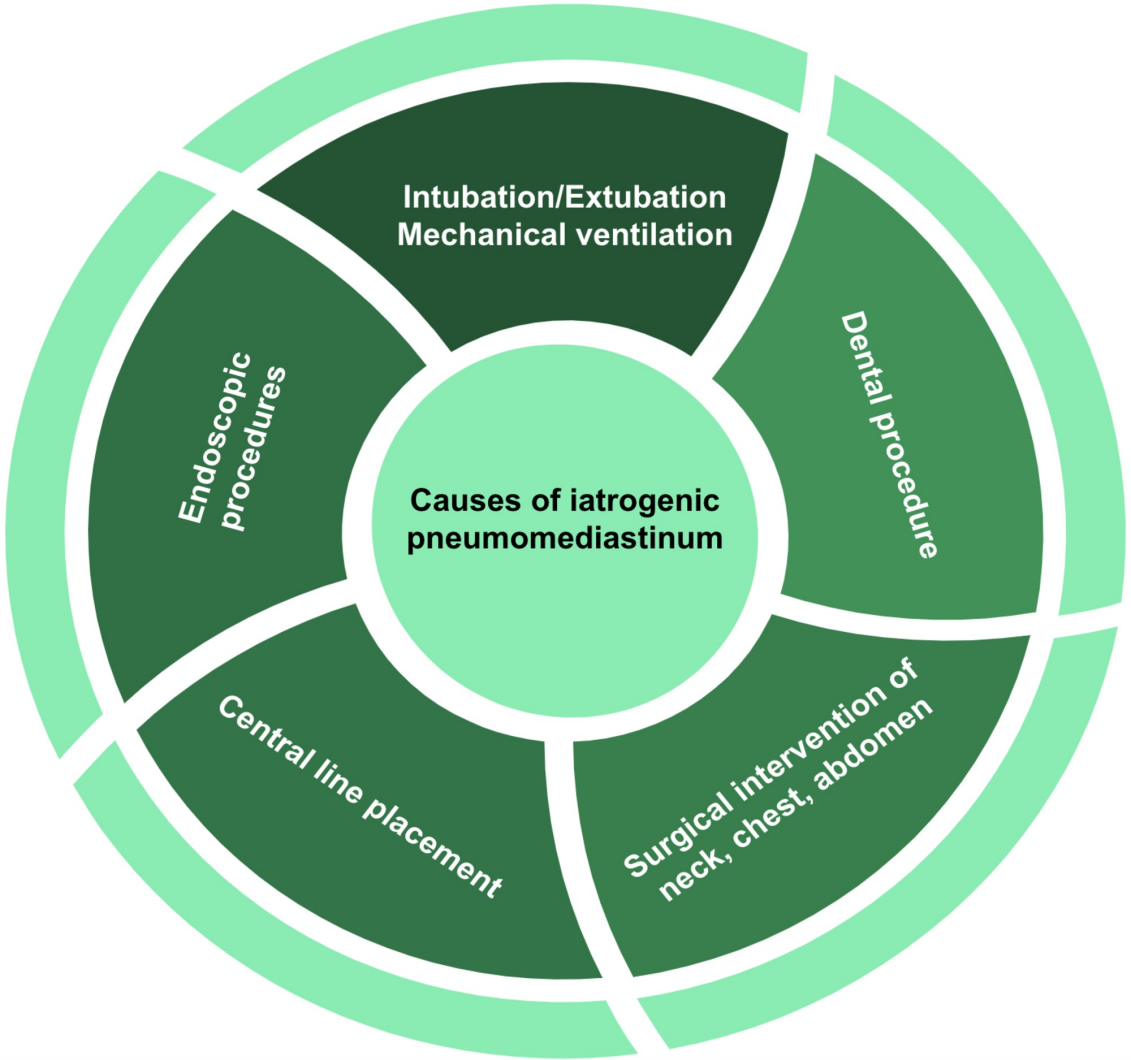
Causes of iatrogenic pneumomediastinum

Pneumomediastinum is dichotomized by etiology, one as a result of blunt force trauma or caused iatrogenically due to endoscopic or other therapeutic procedures, also referred to as secondary PM. The possible causes of iatrogenic PM have been summarized in Figure 5. The second is the presence of free air in the mediastinal cavity without a clear and identifiable etiology [3]. It has been reported that in up to 92% of cases of PM there is a presence of subcutaneous emphysema as was evidenced in our patient [8]. In addition, our patient showed substantial pneumoperitoneum which may be explained by the spaces between the retroperitoneum, mediastinum and subcutaneous tissue being anatomically continuous, and air emerging from a lesion in any of these areas can travel to another place along the fascial planes [9].

Clinical examination of such patients depends on the exact location and presence of the free air. In our patient, subcutaneous emphysema was abundant and physical examination reflected that. In the spontaneous version of PM, Hamman’s crunch was only seen in 18% of patients [3]. Our patient did demonstrate crepitus on the upper chest, both upper extremities as well as the mons pubis and upper thighs. Crepitus is a common feature of subcutaneous emphysema which is usually self-limiting [10]. Our patient was also found to have a distended abdomen in the setting of pneumoperitoneum. In most cases pneumoperitoneum, if not associated with perforation of the alimentary tract, is self-limiting. Of concern is the possibility of abdominal compartment syndrome which may be assessed by determining bladder pressure. Other clinical symptoms were not able to be elicited due to the patient’s intubated state. Common complaints of PM include chest pain, dyspnea, neck pain, cough and rhinolalia [3].

The treatment of presence of free air in body cavities is dependent on location. Our patient had a multifocal presence of free air in the mediastinum, peritoneum and subcutaneous tissue. It has been observed that high-concentration of oxygen has been helpful for subcutaneous and mediastinal emphysema. Pneumothorax, subcutaneous and mediastinal emphysema is reabsorbed into capillaries by diffusion along a partial pressure gradient caused by the sum of partial pressures exerted by water, carbon dioxide, nitrogen and oxygen. With breathing 100% oxygen, nitrogen is washed out of the blood, thus increasing the gradient for gas absorption and causing a four- to six-fold increase in the rate of gas absorption [11, 12]. Our patient was on FiO_2_ of 100% for 48 hours. Repeated radiographs showed improvement in the subcutaneous emphysema, pneumomediastinum. Ventilatory support also plays a role in management. Although there is no ideal target tidal volume or PEEP setting, they should be readjusted to levels as low as is tolerated while simultaneously achieving acceptable gas exchange as high PEEP itself can be a cause of further barotrauma complications as PM, pneumothorax, and extra-thoracic dissection [13, 14]. Other methods have been used to address subcutaneous emphysema. Cases have shown that a 2-3 cm blowhole incision in the supraclavicular or infraclavicular area may be used to eliminate the presence of subcutaneous air. For patients who are on ventilators this may prove challenging as mechanical ventilation leads consistently to the formation of large amounts of air. Negative pressure wound therapy along with blowhole incisions may be used for the treatment of severe SE [15]. Other cases have demonstrated the successful use of subcutaneous drains to effectively decompress the head and neck areas, and markedly reduce airway pressure and subcutaneous air [16].

Complications of PM are dependent on the etiology. In the setting of spontaneous PM complications remain extremely rare as there is a self-limiting course. Iatrogenic or traumatic PM may be associated with other free-air pathologies such as pneumothorax and pneumopericardium with a small chance to convert to tension or 'malignant' PM leading to great vessel compression [17, 18]. Our patient incurred a substantial pneumoperitoneum which could have led to abdominal compartment syndrome [19].

## Conclusions

Tracheostomy and tracheal tubes are commonly used in patients who require prolonged ventilatory support. Though routine and well-practiced techniques they still come with a risk of complication. In our patient, who required long-term mechanical ventilatory support, tracheal mispositioning may have been the culprit in causing PM, pneumoperitoneum as well as massive subcutaneous emphysema. Our patient was treated conservatively with ventilator setting adjustment to increase oxygen deliverance as well as low PEEP pressures. Our case also demonstrates impressive imaging that may be seen in pneumo-pathologies. Unfortunately after 11 days in the hospital, the patient succumbed to her comorbidities. Physicians should be aware of free air pathologies such as PM, pneumoperitoneum and subcutaneous in the differential diagnosis of long-term ventilated patients.
